# Antineutrophil Cytoplasmic Antibody Induction due to Infection: A Patient with Infective Endocarditis and Chronic Hepatitis C

**DOI:** 10.1155/2016/3585860

**Published:** 2016-03-21

**Authors:** Fareed B. Kamar, T. Lee-Ann Hawkins

**Affiliations:** ^1^Department of Medicine, University of Calgary, Calgary, AB, Canada T2N 4N1; ^2^Department of Medicine, Division of General Internal Medicine, University of Calgary, Calgary, AB, Canada T2N 4N1

## Abstract

While antineutrophil cytoplasmic antibody (ANCA) is often used as a diagnostic marker for certain vasculitides, ANCA induction in the setting of infection is much less common. In the case of infective endocarditis, patients may present with multisystem disturbances resembling an autoimmune process, cases that may be rendered even trickier to diagnose in the face of a positive ANCA. Though not always straightforward, distinguishing an infective from an inflammatory process is pivotal in order to guide appropriate therapy. We describe an encounter with a 43-year-old male with chronically untreated hepatitis C virus infection who featured ANCA positivity while hospitalized with acute bacterial endocarditis. His case serves as a reminder of two of the few infections known to uncommonly generate ANCA positivity. We also summarize previously reported cases of ANCA positivity in the context of endocarditis and hepatitis C infections.

## 1. Introduction

The antineutrophil cytoplasmic antibody (ANCA) class of immunoglobulins features the principal subtypes c-ANCA and p-ANCA, which are predominantly generated against the cytosolic antigens proteinase 3 (PR3) and myeloperoxidase (MPO), respectively [1]. The presence of these autoantibodies has been described in a variety of autoimmune conditions, such as small-vessel vasculitides, ulcerative colitis, primary sclerosing cholangitis, and autoimmune hepatitis [2, 3]. Less frequently, ANCA induction can occur due to infections such as amebiasis, endocarditis, tuberculosis, malaria, human immunodeficiency virus infection, and hepatitis C virus (HCV) infection [2, 4]. Because autoimmune and infectious diseases may present similarly, ANCA positivity must be carefully interpreted [5]. The following case describes a 43-year-old male with chronically untreated HCV infection who was admitted to hospital with infective endocarditis and was found to be c-ANCA positive. We also summarize the literature concerning ANCA positivity in endocarditis and HCV infections.

## 2. Clinical Vignette

**Figure 1:**
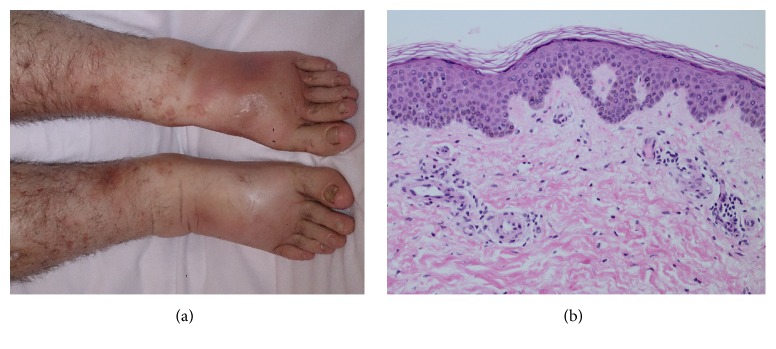
(a) Photograph of the patient's resolving purpura involving his legs one week into antibiotic therapy. (b) Corresponding hematoxylin and eosin-stained histopathology at 20x magnification of a punch biopsy of one of the lesions on his leg, showing mild perivascular inflammation with focally extravasated erythrocytes consistent with a resolving purpuric process. No leukocytoclastic vasculitis was seen.

A 43-year-old male with a history of HCV infection (untreated since his diagnosis six years previously, with an RNA viral load of 1584 IU/mL on admission) and intravenous polysubstance use presented to a medical center with acute fever, dyspnea, and arthralgia. He was found to have purpura over his edematous lower extremities. His initial laboratory investigations featured an elevated white blood cell count of 16 × 10^9^ cells per liter, elevated C-reactive protein of 183 mg/L, urinalysis that was positive for hematuria, and blood cultures that were later positive for methicillin-sensitive* Staphylococcus aureus*. He did feature transient acute kidney injury soon after admission (peak serum creatinine 283 umol/L). His serology was also positive for c-ANCA (anti-PR3), antinuclear antibody, and weakly positive for type III cryoglobulinemia. An echocardiogram revealed 1.1 × 1.3 cm tricuspid vegetation involving the anterior and septal leaflets. A computerized tomography scan of his chest illustrated multiple bilateral septic pulmonary emboli, bilateral pleural effusions, and an anterior mediastinal abscess. A punch biopsy of a purpuric lesion on his right shin, performed one week into antibiotic therapy as his purpura was resolving (Figure 1(a)), revealed mild perivascular inflammation with focally extravasated erythrocytes and hemosiderin deposits consistent with a mild or resolving purpuric process (Figure 1(b)). The patient demonstrated clinical improvement during a six-week course of cefazolin.

## 3. Discussion

ANCA positivity may pose a diagnostic and therapeutic quandary in the face of patient presentation consistent with either a vasculitic or infectious process, particularly in the case of infective endocarditis [6].

A literature search of previously published cases concerning ANCA induction in infective endocarditis was performed via PubMed and Medline using the title and abstract entries “endocarditis” and “ANCA or antineutrophil cytoplasmic antibody,” yielding 70 relevant cases [1, 5–39]. A recent publication by Ying et al. (2014) describes 13 of these cases in addition to a literature review of several other ones [7]. We have expanded on this review through the addition of 26 other cases (Table 1).

A set of diagnostic aids between infective endocarditis and small-vessel vasculitis has been previously outlined (Table 2) [8]. One similarity, for example, is acute renal failure, the prevalence of which in bacterial endocarditis is 30% and is a significant predictor of mortality [40]. Glomerulonephritis in infective endocarditis is either pauci-immune, postinfective, or subendothelial membranoproliferative [41], the etiology of which can usually be discerned by obtaining a kidney biopsy [8].

Another ANCA-associated infection present in our reported patient is HCV infection. Previously published cases of ANCA induction due to hepatitis C infection are also summarized (Table 3) [6, 9, 42–54]. Our case report hence features two possible infections for c-ANCA induction, both of which likely also contributed to the patient's cryoglobulinemia. Because ANCA induction is more common in chronic infections [6], it argues for hepatitis C as the cause of this patient's ANCA positivity as opposed to the more acute infection* Staphylococcus aureus* endocarditis [55]. Had his ANCA status been checked after endocarditis recovery, ANCA induction due to endocarditis as opposed to hepatitis C would have also been supported by a normalized or negative ANCA titer [11].

## 4. Conclusion

In light of its use in the diagnostic evaluation of vasculitis, a positive ANCA may allow for an infection to mislead a diagnostician down the path of autoimmune possibilities, particularly in the context of infective endocarditis. While clues may be drawn from clinical, laboratory, and radiological data to help differentiate infective endocarditis from vasculitis, obtaining blood cultures is of foremost importance. Making such a distinction will avoid the detrimental consequence of initiating immunosuppressive therapy against an infection masquerading as an inflammatory disease.

## Figures and Tables

**Table 1:** Number of positive clinical and laboratory characteristics among all previously reported cases of ANCA-positive infective endocarditis^*∗*^.

Patient characteristic	Proportion among 70 reported patient cases
Mean age (years)	53.2
Male/female	54/16
Valve involvement	56/70
Aortic	22
Mitral	16
Left-sided not otherwise specified	7
Aortic plus mitral	6
Tricuspid	5
Pulmonary	1
Mitral plus pulmonary and tricuspid	1
Ventricular septal defect	1
Clinical features	
Fever	46
Anemia	34
Splenomegaly	19
Nephropathy (GN or AKI)	43
Arthralgia	17
Lower extremity edema	23
Rash	15
Purpura	11
Cerebral infarction	7
Finger clubbing	4
Laboratory results	
PR3	52
MPO	8
PR3 + MPO	7
Hematuria	49
Proteinuria	14
Microbiology	
Positive blood culture	54/70
Pathogen	
*Streptococcus *spp.	28
*Enterococcus *spp.	7
*Staphylococcus *spp.	10
*Bartonella *spp.	9
*Neisseria *spp.	1
*Propionibacterium *spp.	1
*Haemophilus *spp.	1
*Gemella *spp.	1
*Aggregatibacter *spp.	1

GN: glomerulonephritis; AKI: acute kidney injury; PR3: proteinase 3; MPO: myeloperoxidase; spp.: species.

^*∗*^This table, taken from Ying et al. (2014) with permission, expands the review from the original 44 patients to include 26 others [7].

**Table 2:** Diagnostic aids for differentiating between infectious endocarditis and small-vessel vasculitis^*∗*^.

Similarities^a^	Differences^b^
(i) Presentation with constitutional symptoms	(i) Splenomegaly
(ii) Pyrexia	(ii) Thrombocytopenia
(iii) Active urinary sediment	(iii) Hypocomplementemia
(iv) Skin involvement	(iv) Immune complexes
(v) Decreased GFR	(v) Other positive autoantibodies
(vi) Increased inflammatory marker levels	(vi) Low titer ANCA/ELISA negative
	(vii) Other organ involvement

ANCA: antineutrophil cytoplasmic antibody; ELISA: enzyme-linked immunosorbent assay; GFR: glomerular filtration rate.

^a^Features seen in both conditions.

^b^Features seen predominantly in infectious endocarditis.

^*∗*^This table was taken from Forbes et al. (2012) with permission [8].

**Table 3:** Summary of previously published ANCA-positive hepatitis C infection cases.

Paper	Age (years), sex	ANCA	Miscellaneous features
Bonaci-Nikolic et al., 2010 [6]	63, F	MPO	—
	51, F	MPO	—
	24, F	MPO	—

Cojocaru et al., 2007 [42]	Mean 75	21 PR3	Concomitant ischemic stroke

Cojocaru et al., 2006 [43]	?	?	—

Gatselis et al., 2006 [44]	?	65 c-ANCA, 4 p-ANCA (though all negative for PR3 and MPO)	

Lamprecht et al., 2003 [9]	?	6 bactericidal/permeability-increasing proteins	Mixed cryoglobulinemia
		4 cathepsin G proteins	
		1 unknown antigen (c-ANCA)	
		2 bactericidal/permeability-increasing proteins	No cryoglobulinemia
		Four patients: cathepsin G	

Zandman-Goddard et al., 2003 [45]	34, M	c-ANCA and p-ANCA	Complicated by transverse myelitis

Tajima et al., 2002 [46]	66, F	p-ANCA	Complicated by pachymeningitis

Wu et al., 2002 [47]	?	253 PR3, 25 PR3 and MPO	Higher proportion of ANCA-positive compared to ANCA-negative patients with high alanine aminotransferase, high alpha-fetoprotein, skin disease, cirrhosis, and anemia

Agarwal et al., 2001 [48]	?	5 p-ANCA	—

Igaki et al., 2000 [49]	60, F	MPO	Glomerulonephritis, cryoglobulinemia

Lamprecht et al., 1998 [50]	60, F	c-ANCA	Type II cryoglobulinemia

Ohira et al., 1998 [51]	?	12 c-ANCA or p-ANCA	—

Kallinowski et al., 1997 [52]	?	5 ANCA	—

Papi et al., 1997 [53]	63, F	MPO	Mixed type II cryoglobulinemia, leukocytoclastic vasculitis on skin biopsy

Dalekos and Tsianos, 1994 [54]	?	3 ANCA	—

F: female; ANCA: antineutrophil cytoplasmic antibody; MPO: myeloperoxidase; PR3: proteinase 3.
